# Retraction Note: Runt-related transcription factor 1 promotes apoptosis and inhibits neuroblastoma progression in vitro and in vivo

**DOI:** 10.1186/s13046-025-03581-7

**Published:** 2025-11-22

**Authors:** Mei Hong, Jing He, Duo Li, Yuanyuan Chu, Jiarui Pu, Qiangsong Tong, Harish C. Joshi, Shaotao Tang, Shiwang Li

**Affiliations:** 1 Department of Pediatric Surgery, Union Hospital, Tongji Medical College, Huazhong University of Science and Technology, Wuhan, 430022 China; 2 Institute of Hematology, Union Hospital, Tongji Medical College, Huazhong University of Science and Technology, Wuhan, China; 3 Central Laboratory, Union Hospital, Tongji Medical College, Huazhong University of Science and Technology, Wuhan, China; 4 Department of Cell Biology, Emory University, Atlanta, GA USA

**Retraction Note: J Exp Clin Cancer Res 39, 52 (2020)**

**https://doi.org/10.1186/s13046-020–01558-2**

The Editor-in-Chief has retracted this article. After publication, a number of image integrity concerns were raised. Specifically:

- two panels in Fig. 2d appear to overlap with frameshift
- a panel in Figs. 3b and 7f appear to overlap
- two panels in Fig. 6c appear to overlap

The authors did not provide their original images upon request. The Editor has lost confidence in the data and conclusions of this article.

Author Mei Hong disagrees with this retraction. All other authors did not respond to correspondence from the Publisher about this retraction.

